# Proteomic Analysis of the Hydrogen and Carbon Monoxide Metabolism of *Methanothermobacter marburgensis*

**DOI:** 10.3389/fmicb.2016.01049

**Published:** 2016-07-04

**Authors:** Martijn Diender, Ricardo Pereira, Hans J. C. T. Wessels, Alfons J. M. Stams, Diana Z. Sousa

**Affiliations:** ^1^Laboratory of Microbiology, Wageningen UniversityWageningen, Netherlands; ^2^Department of Laboratory Medicine, Radboud University Medical CenterNijmegen, Netherlands; ^3^Centre of Biological Engineering, University of MinhoBraga, Portugal

**Keywords:** syngas, methanogenesis, CODH, reductive acetyl-CoA pathway, *Methanothermobacter thermoautotrophicus*

## Abstract

Hydrogenotrophic methanogenic archaea are efficient H_2_ utilizers, but only a few are known to be able to utilize CO. *Methanothermobacter thermoautotrophicus* is one of the hydrogenotrophic methanogens able to grow on CO, albeit about 100 times slower than on H_2_ + CO_2_. In this study, we show that the hydrogenotrophic methanogen *Methanothermobacter marburgensis*, is able to perform methanogenic growth on H_2_/CO_2_/CO and on CO as a sole substrate. To gain further insight in its carboxydotrophic metabolism, the proteome of *M. marburgensis*, grown on H_2_/CO_2_ and H_2_/CO_2_/CO, was analyzed. Cultures grown with H_2_/CO_2_/CO showed relative higher abundance of enzymes involved in the reductive acetyl-CoA pathway and proteins involved in redox metabolism. The data suggest that the strong reducing capacity of CO negatively affects hydrogenotrophic methanogenesis, making growth on CO as a sole substrate difficult for this type of methanogens. *M. marburgensis* appears to partly deal with this by up-regulating co-factor regenerating reactions and activating additional pathways allowing for formation of other products, like acetate.

## Introduction

Methanogenesis from hydrogen, acetate, methanol, or methanethiols is a relatively well-studied process (Thauer et al., 2008). In 1931, CO was shown to potentially act as a substrate for methanogens in mixed cultures (Fischer et al., 1931), but knowledge of carboxydotrophic methanogenesis is still rather limited. Gaining insight in CO-driven methanogenesis is not only of interest from a fundamental point of view, but also from an applied scope. The rapid development of syngas technology makes it cheaper to convert any carbon rich source into a gaseous mixture, consisting mainly of CO, H_2_, and CO_2_ (syngas). Syngas-driven carboxydotrophic methanogenesis can be considered as an alternative to biogas production via anaerobic digestion. About 10–25% of the biomass consists of lignin, which is difficult to degrade biologically and can even prevent degradation of easily degradable biopolymers such as hemicellulose (Daniell et al., 2012; Jönsson and Martín, 2016). Additionally, biomass-derived biogas contains a fraction of 25–45% CO_2_ (De Mes et al., 2003) and needs to be subsequently upgraded to bio-methane before injection in the gas grid is possible. Via the syngas route a higher substrate conversion yield can be achieved, and by increasing the H_2_ content of the gas substrate a CO_2_-free end product can be obtained in one process step.

Carbon monoxide appears to be a difficult substrate for methanogens, and only three methanogens have been shown to utilize it for growth: *Methanothermobacter thermoautotrophicus* (Daniels et al., 1977), *Methanosarcina barkeri* (O’Brien et al., 1984; Bott et al., 1986), and *Methanosarcina acetivorans* (Rother and Metcalf, 2004). Carboxydotrophic growth of *M. thermoautotrophicus* is possible with CO as a sole carbon source up to pressures of 50 kPa. Doubling times reported for CO-grown *M. thermoautotrophicus* are about 200 h, which is about a 100 times slower than with H_2_/CO_2_ (Daniels et al., 1977). *M. barkeri* was observed to utilize 100 kPa CO, but also at a relatively slow doubling time (about 65 h) compared to growth on other substrates (O’Brien et al., 1984). *Methanosarcina acetivorans* can withstand higher CO partial pressures (>150 kPa) and grows on CO with a doubling time of about 20 h. However, it shifts its methanogenic metabolism toward formation of acetate and formate with increasing CO pressures (Rother and Metcalf, 2004). Detailed research has been performed on the CO metabolism of *M. acetivorans* (Rother and Metcalf, 2004; Lessner et al., 2006; Rother et al., 2007; Matschiavelli et al., 2012), providing more insight in the enzymes involved in carboxydotrophic metabolism of this aceticlastic methanogen. Currently, *M. thermoautotrophicus* is the only hydrogenotrophic methanogen known that can grow on CO as a substrate, and there is limited knowledge on the carboxydotrophic metabolism of hydrogenotrophic methanogens in general. Carboxydotrophic methanogenesis is expected to be operated in a similar way as hydrogenotrophic metabolism (Daniels et al., 1977; Diender et al., 2015). Electrons derived from the oxidation of CO or H_2_ are used to reduce CO_2_ to methyl-tetrahydromethanopterin (methyl-H_4_MPT). This key-intermediate can subsequently be converted into either methane, for energy generation, or assimilated via acetyl-CoA, for biomass production.

In this study, we aimed to assess the CO-metabolism of *Methanothermobacter marburgensis*. *M. marburgensis* has a highly similar CODH sequence to that of *M. thermoautotrophicus* (93% identity). Additionally, it was shown that the methyl-coenzyme M reductase (MCR) of *M. marburgensis* was activated 15 times faster by CO than by H_2_ (Zhou et al., 2013). Here we show that *M. marburgensis* can grow methanogenically on CO and we assessed its carboxydotrophic metabolism via physiological analysis and proteomics. Additionally, we evaluated the limitations of this type of metabolism, which is theorized to be related to hydrogenase inhibition or redox-stress (Diender et al., 2015).

## Materials and Methods

### Strains and Cultivation

Strains *M. thermoautotrophicus* (DSM 1053) and *M. marburgensis* (DSM 2133) were obtained from the German Collection of Microorganisms and Cell Cultures (DSMZ; Braunschweig, Germany). Strains were initially cultivated at 65°C on recommended *Methanobacterium* medium (DSM-119) using anaerobic cultivation procedures. After growth was confirmed, the strains were adapted to growth in a carbonate-phosphate buffered medium with the following composition: 0.4 g/l KH_2_PO_4_, 0.53 g/l K_2_HPO_2_^∗^2 H_2_O, 0.3 g/l NH_4_Cl, 0.3 g/l NaCl, 0.1 g/l MgCl_2_^∗^6 H_2_O, 0.5 g/l yeast extract and 0.5 mg/l resazurin. Medium was supplemented, per liter, with 61.8 μg H_3_BO_3_, 61.25 μg MnCl_2_, 943.5 μg FeCl_2_, 64.5 μg CoCl_2_, 12.86 μg NiCl_2_, 67.7 μg ZnCl_2_, 13.35 μg CuCl_2_, 17.3 μg Na_2_SeO_3_, 29.4 μg Na_2_WO_4_, and 20.5 μg Na_2_MoO_4_. Medium was prepared, boiled and cooled under a continuous nitrogen flow. Bottles (120 ml total volume, 50 ml liquid) were filled with medium and instantly capped with rubber stopper and aluminum cap. The gas phase was exchanged with either 80:20 N_2_/CO_2_ or 80:20 H_2_/CO_2_, resulting in a final pressure of 170 kPa. When necessary, the headspace was further fine-tuned by addition of CO, keeping final pressure at 170 kPa. The bottles were autoclaved and stored at room temperature till further use. Before inoculation medium was supplied with the following volumes of stock solutions: 1% of 11 g/l CaCl_2_^∗^2 H_2_O, 1% of a vitamin solution containing per liter: biotin 20 mg, nicotinamide 200 mg, *p*-aminobenzoic acid 100 mg, thiamin 200 mg, panthotenic acid 100 mg, pyridoxamine 500 mg, cyanocobalamin 100 mg, riboflavin 100 mg. The medium was reduced by introducing a 5% volume of a stock solution containing 4.8 g/l Na_2_S ^∗^ *x* H_2_O (*x* = 7 - 9) and 80 g/l NaHCO_3_. Unless stated otherwise, bottles were inoculated with an exponentially growing culture in a 1:50 ratio (v/v).

### Analytical Procedures

Headspace composition was determined by gas chromatography using a GC-2014 (Shimadzu, Kyoto, Japan) equipped with a thermal conductivity detector. H_2_, CH_4_, and CO were measured with a Molsieve 13X column, 2 m long and an inner diameter of 3 mm. Argon was used as carrier gas at a flow rate of 50 ml/min. Injector, column and detector temperatures were set to 80, 60, and 130°C, respectively. CO_2_ was measured separately in a CP Poraplot column of 25 m × 0.53 mm, with a stationary phase film thickness of 20 μm, employing helium as carrier gas at a flow rate of 15 ml/min. Injector, column and detector temperatures were set to 60, 34, and 130°C, respectively. The detection limits for H_2_, CO, CO_2_, and CH_4_ were 20, 250, 20, and 80 Pa, respectively.

Liquid phase composition was analyzed via high pressure liquid chromatography equipped with a MetaCarb 67H column (Agilent Technologies, Santa Clara, CA, USA). The column was operated at a temperature of 45°C with a flow rate of 0.8 ml/min using 0.01 N H_2_SO_4_ as eluent. Detection of acetate was done using an RI and UV detector. Concentrations of 0.5 mM could be accurately determined and lower levels are referred to as trace amounts.

### Cell Free Extract Preparation and Measuring CO-Oxidation Activity

Cells of *M. marburgensis* were grown on either 80:20 H_2_/CO_2_ or 60:20:20 H_2_/CO_2_/CO. Biological triplicates were prepared for each condition. Cells were harvested at end log-phase in an anaerobic tent, where the broth was centrifuged at 13000 × *g* for 10 min. Supernatant was discarded and the pellets were dissolved in 1 ml 50 mM Tris-HCl, pH 8. Cells were disrupted using a VC-40 sonicator (Sonics materials, Newtown, CT, USA) using five cycles of 30 s sonication, at a power input of 20 W, followed by 30 s rest at 0°C. Cell free extract (CFE) was put in 5 ml glass vials and capped with rubber stoppers inside the anaerobic tent. Subsequently, the headspace of these vials was exchanged to 1.5 bar N_2_ to remove traces of H_2_.

CO-oxidation activity of the CFE was determined as follows: glass cuvettes (2 ml total volume) were closed with a rubber stopper and flushed three times by using 5 ml N_2_ or CO. Subsequently, 1 ml assay buffer (50 mM MOPS, pH 7, 20 mM methyl viologen (MV) and 2 mM DTT) was added to the cuvette and reduced with 1 μl of 100 mM dithionite. The cuvette was put into a pre-heated U-2010 spectrophotometer (Hitachi, Tokyo, Japan) and was left to heat up to 60°C. Absorption of MV was measured at 578 nm. The initial extinction was set to a value between 0.2 and 0.6. After obtaining a stable baseline, 50 or 25 μl CFE was added to the cuvette, initiating the reaction. Each separate biological sample was assessed for CO-oxidizing activity and endogenous activity (using N_2_ instead of CO). The initial activity of CO-oxidation after CFE addition was taken, and corrected for the initial slope of the endogenous activity. In order to confirm that CO was the electron donor, cuvettes were prepared with N_2_ headspace and CFE was added. After seizure of endogenous activity 0.3 ml CO was added to the sample as initiation trigger. Each biological sample was at least analyzed in duplicate using either 50 or 25 μl CFE. An extinction coefficient 9.7 mM^-1^ cm^-1^ (Bonam and Ludden, 1987) was used for MV at 578 nm. Protein concentration in the CFE was determined by using Roti-Nanoquant protein quantitation assay (CarlRoth, Karlsruhe, Germany), according to manufacturer instructions.

### Sample Preparation for Proteomics

Duplicate cultures of *M. marburgensis* grown in two conditions, 80:20 H_2_/CO_2_ and 60:20:20 H_2_/CO_2_/CO, were harvested in late exponential phase by centrifugation (cultivation was performed in 1 l anaerobic bottles containing 500 ml medium). Prior to centrifugation cultures were quickly cooled down on ice and kept at 4°C for 30 min to decrease cell activity. Cell pellets were resuspended in TE buffer (10 mM Tris-Cl, pH 7.5; 1 mM EDTA) containing 1 mM phenylmethanesulfonyl fluoride, and passed through a French pressure cell operated at 138 MPa. Proteins were stabilized by addition of 8 M of urea in a proportion of 1:1 and samples were concentrated using a 3.5 kDa MWCO filter. Final protein concentration in samples obtained for LC-MS/MS analysis were assessed using Qubit^®^ Protein Assay Kit in a Qubit^®^ 2.0 Fluorometer (Life technologies). Samples were subjected to in-solution tryptic digestion as described elsewhere (Wessels et al., 2010).

### LC-MS/MS Data Acquisition

Protein samples obtained from the two sets of biological duplicates were analyzed in duplicate using C18 reversed phase liquid chromatography with online tandem mass spectrometry (LC-MS/MS). Measurements were performed using a nanoflow ultra-high pressure liquid chromatograph (nano-Advance; Bruker Daltonics) coupled online to an orthogonal quadrupole time-of-flight mass spectrometer (maXis 4G ETD, otofControl v3.4 build 14; Bruker Daltonics) via an axial desolvation vacuum assisted electrospray ionization source (Captive sprayer; Bruker Daltonics). Five microliters of tryptic digest were loaded onto the trapping column (Acclaim PepMap 100, 75 μm × 2 cm, nanoViper, 3 μm 100Å C18 particles; Thermo Scientific) using 0.1% FA at a flow rate of 9000 nl/min for 3 min at room temperature. Next, peptides were separated on a C18 reversed phase 15 cm length × 75 μm internal diameter analytical column (Acclaim PepMap RSLC, 75 μm × 15 cm, nanoViper, 2 μm 100Å C18 particles; Thermo scientific) at 40°C using a linear gradient of 3–35% ACN 0.1% FA in 120 min at a flow rate of 600 nl/min. The mass spectrometer was operated in positive ion mode and was tuned for optimal ion transmission in the range of m/z 300–1400. Electrospray ionization conditions were 3 l/min 180°C N_2_ drying gas, 1400 V capillary voltage and 0.4 Bar N_2_ for gas phase supercharging (nanobooster) using acetonitrile as dopant. Parameters for optimal ion transmission were funnel RF: 400 Vpp, multipole RF: 400 Vpp, quadrupole ion energy: 5.0 eV, quadrupole low mass: 300 m/z, collision cell energy: 9.0 eV, collision cell RF: 3500 Vpp, ion cooler transfer time: 64 μs, ion cooler RF: 250 Vpp, pre-pulse storage: 22 μs. Data dependent acquisition of MS/MS spectra (AutoMSn) was performed using a 3 s duty cycle at 2 Hz acquisition rate for full MS spectra and a variable number of MS/MS experiments at precursor intensity scaled spectra rate (3 Hz MS/MS spectra rate at 2000 counts, 20 Hz MS/MS spectra rate @ 100.000 counts). Precursor ions within the range of 400–1400 m/z with charge state *z* = 2+ or higher (preferred charge state range of *z* = 2+ to *z* = 4+) were selected for MS/MS analysis with active exclusion enabled (excluded after one spectrum, released after 0.5 min, reconsidered precursor if current intensity/previous intensity ≥4, smart exclusion disabled). Spectra were saved as line spectra only and were calculated from profile spectra as the sum of intensities across a mass spectral peak (five counts absolute threshold, peak summation width seven points).

### Proteomics Analysis

Protein identification and relative quantitation was performed using the MaxQuant software (v.1.5.0.0; Cox and Mann, 2008) using the build-in Andromeda database search algorithm. Extracted MS/MS spectra were searched against the SWISS-PROT *M. marburgensis* protein sequence database. Amino acid sequences of known contaminant proteins (e.g., skin and hair proteins, Trypsin, LysC) were added to the database. The following settings were used for peptide and protein identification: carbamidomethyl (Cys) as fixed modification, oxidation (Met), and deamidation (NQ) as variable modifications, predefined MS and MS/MS settings for TOF instruments, minimal peptide length six amino acids and a maximum allowed false discovery rate of 1% at both the peptide and protein level. Label free quantitation (LFQ) was performed with the match between runs and re-quantify options using at least 2 razor + unique peptides. Retention time alignment was performed with a time alignment window of 20 min and a retention time match window of 0.5 min. Label-free quantitation (LFQ) values were used for subsequent data analysis. Proteins quantified in at least three out of four measurements for either growth condition were analyzed by student’s *t*-tests to identify differentially expressed proteins with *p* < 0.05. The mass spectrometry proteomics data have been deposited to the ProteomeXchange Consortium via the PRIDE (Vizcaíno et al., 2016) partner repository with the dataset identifier PXD003661.

## Results

### Carboxydotrophic Growth of *M. thermoautotrophicus* and *M. marburgensis*

**FIGURE 1:**
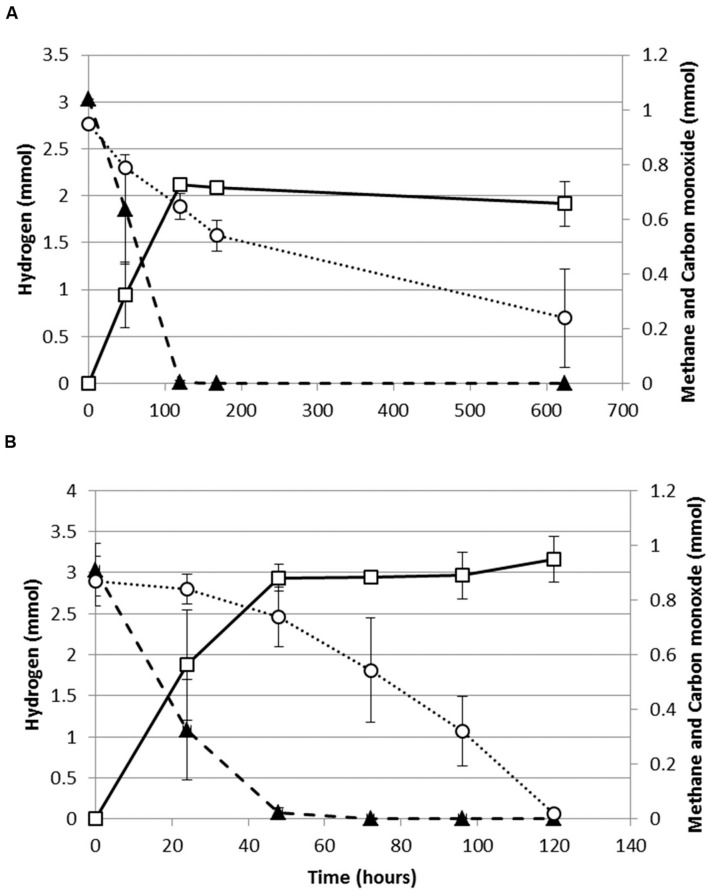
**Production profile of hydrogenotrophic methanogens growing on H_2_/CO_2_/CO.** **(A)** Production profile of *M. thermoautotrophicus*. **(B)** Production profile of *M. marburgensis*. Hydrogen: solid black triangles, Methane: open black squares, Carbon monoxide: open black circles. Gas is represented as total amount of mmol present in the bottle headspace. Error bars display maximal and minimal amounts over duplicate experiments.

Both, *M. thermoautotrophicus* and *M. marburgensis* were capable of growing methanogenically on H_2_/CO_2_/CO or CO as a sole substrate. When assessing the production profiles on H_2_/CO_2_/CO, it appears that both strains co-utilize H_2_ and CO (**Figure 1**). For both strains H_2_ utilization becomes slower with exposure to higher CO pressures. The maximal H_2_ consumption rate of *M. marburgensis* dropped from 1.3 to 0.43 mmol/l_liquid_/h when increasing the CO pressure from 0 to 70 kPa. Similarly *M. thermoautotrophicus* decreased its maximal H_2_ consumption rate from 0.55 to 0.19 mmol/l_liquid_/h when increasing the CO pressure from 0 to 70 kPa. *M. marburgensis* could be grown on CO as a sole substrate up to 50 kPa (data not shown), which is similar to the value found for *M. thermoautotrophicus* (Daniels et al., 1977).

**FIGURE 2:**
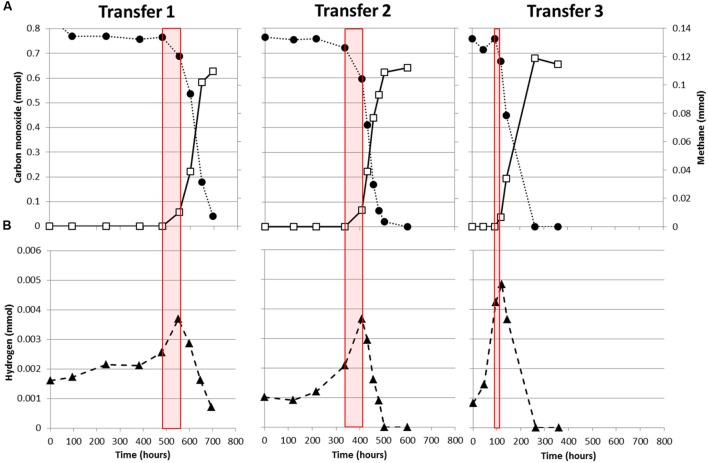
**Adaptation of *M. marburgensis* to carboxydotrophic growth.** **(A)** Consumption of CO (black solid circles) and production of methane (open squares). **(B)** H_2_ production and consumption profile during growth on CO. Red planes indicate the timeframe where methanogenesis is initiated. Gas is represented as total amount of mmol present in the bottle headspace.

*M. marburgensis* appears to utilize carbon monoxide more easily as it depletes 34 kPa CO within 120 h, while *M. thermoautotrophicus* uses this substrate significantly slower (**Figure 1**). This suggests that *M. marburgensis* can be better adapted to carboxydotrophic growth than *M. thermoautotrophicus*. It was attempted to adapt both strains to cultivation on purely CO by repeatedly transferring them in presence of solely CO as electron donor. The initial lag phase of *M. marburgensis* growing solely on 34 kPa CO was ∼500 h, but in the two subsequent transfers the lag phase for CO conversion decreased to ∼100 h (**Figure 2**). CO consumption and methane production rates between these transfers did not change significantly (**Figure 2**). Cultures transferred back to H_2_/CO_2_ quickly lost this adaptation to CO and showed again a longer lag phase when incubated with CO alone. Nevertheless, a re-adaptation of these cultures to CO was possible. Growth of *M. thermoautotrophicus* on H_2_/CO_2_/CO or CO as the sole substrate could not be improved via subsequent transfers, nor could the lag phase be decreased. Due to this inefficient growth of *M. thermoautotrophicus* in presence of CO it was decided to perform further analysis and proteomics solely with *M. marburgensis*.

In all cultures of *M. marburgensis* growing on CO as a sole substrate, H_2_ is observed to accumulate in the headspace before the onset of methanogenesis (**Figure 2**). Upon initiation of carboxydotrophic methanogenesis, H_2_ is co-utilized. At the end of cultivation, not all the CO is converted to methane as approximately 1 mol methane is formed per 6 mol CO consumed (**Figure 2**), suggesting formation of other products. Acetate formation was observed in incubations of *M. marburgensis* grown in presence of CO, which ranged from 2 to 4 mM at the end of cultivation.

Assessing the *M. marburgensis* CO oxidizing activity in the CFE of H_2_/CO_2_/CO grown cells resulted in a specific MV reducing activity of 2.74 ± 0.3 μmol MV min^-1^mg protein^-1^ after correction for endogenous activity. H_2_/CO_2_ grown cells showed a specific MV reducing activity of 2.06 ± 0.13 μmol MV min^-1^mg protein^-1^ after correction for endogenous activity, which is about 1.3 times lower than in the condition with CO. Additionally, the CFE of H_2_/CO_2_ grown cells showed no endogenous activity, while cells grown on H_2_/CO_2_/CO showed an average initial endogenous activity of 0.6 μmol MV min^-1^mg protein^-1^. This endogenous activity decayed over time, completely seizing after ∼150 s. Addition of CO, after seizure of endogenous activity, resulted again in reduction of MV in all samples.

### Comparative Proteomics of *M. marburgensis*

Comparative proteomic analysis was performed on *M. marburgensis* incubated with 80:20 H_2_/CO_2_ or 60:20:20 H_2_/CO_2_/CO. In the proteomics analysis 5845 peptides from 831 non-redundant proteins were identified [false discovery rate (FDR) ≤ 1%, average absolute mass error: 1.14 ± 1.76 ppm) of which 590 proteins were quantified using at least two razor + unique peptides in ≥3 measurements of either growth condition. Both the technical and biological reproducibility was very good based on median LFQ standard deviations of 6.9 and 18.4%, respectively. In total, 203 proteins were found to be differentially abundant between the two growth conditions (student’s *t*-test *p* < 0.05, median LFQ standard deviation: 14.8%).

**FIGURE 3:**
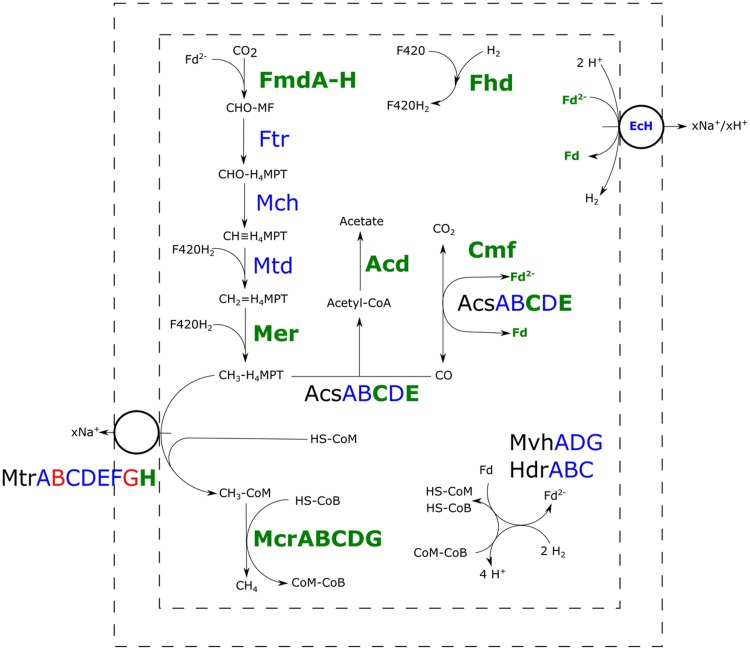
**Comparative proteomic analysis of methanogenic metabolism of *M. marburgensis* grown on H_2_/CO_2_/CO or H_2_/CO_2_.** Relative abundance of proteins of carboxydotrophic growth compared to hydrogenotrophic growth is shown. Proteins highlighted green are found more abundantly present, proteins highlighted blue are not significantly changed in abundance and proteins highlighted in red are found to be less abundant in presence of CO (*p* < 0.05). Fmd, formyl-methanofuran dehydrogenase; Ftr, tetramethanopterin formyl-transferase; Mch, methenyltetramethanopterin cyclohydrolase; Mtd, methylene-H_4_MPT dehydrogenase; Mer, methylene-H_4_MPT reductase; Acs, acetyl-CoA synthase; Acd, acetyl-CoA synthetase; Cmf, CODH maturation factor; Mvh/Hdr, F420-non-reducing hydrogenase/heterodisulfide reductase; Mtr, tetrahydromethanopterin *S*-methyltransferase; Mcr, methyl-coenzyme M reductase; EcH, energy conserving hydrogenase; Fhd, F420 dehydrogenase; H_4_MPT, tetrahydromethanopterin; MF, methanofuran.

All the proteins required for hydrogenotrophic methanogenesis and the reductive acetyl-CoA pathway could be detected in both the H_2_/CO_2_- and H_2_/CO_2_/CO-grown cultures (**Figure 3**). In presence of CO, several subunits of carbon monoxide dehydrogenase (CODH) and acetyl-CoA synthase (ACS) were more abundant. Several enzymes in the methyl-branch of the acetyl-CoA pathway were found to be increased in abundance in the presence of CO (**Table 1**): mainly the formylmethanofuran dehydrogenase, methyl-coenzyme M reductase-I and the F420 dependent methylene-H_4_MPT dehydrogenase. Additionally, a higher abundance of the subunit H of the tetrahydromethanopterin *S*-methyltransferase was detected with CO as substrate. Other subunits of this complex were not significantly overproduced, and levels of subunit B and G were even lowered. Also, a predicted acetyl-CoA synthetase, theorized to be involved in acetate metabolism, was found to be more abundant.

**Table 1:** Highlighted set of more abundant proteins of *M. marburgensis* grown on H_2_/CO_2_/CO vs. H_2_/CO_2_.

Protein	Relative abundance (H_2_/CO_2_/CO vs. H_2_/CO_2_)
**Methanogenesis related proteins**			
Tungsten formylmethanofuran dehydrogenase (subunit A)	2.13
Tungsten formylmethanofuran dehydrogenase (subunit B)	3.16
Tungsten formylmethanofuran dehydrogenase (subunit C)	1.7
Tungsten formylmethanofuran dehydrogenase (subunit D)	2.36
Tungsten Formylmethanofuran dehydrogenase (subunit F)	1.48
Tungsten formylmethanofuran dehydrogenase (subunit G)	2.4
F420-dependent methylene-H4MPT dehydrogenase	1.68
Methyl-coenzyme M reductase component A2-like protein	2.06
Methyl-coenzyme M reductase I subunit alpha	1.97
Methyl-coenzyme M reductase I subunit beta	1.63
Methyl-coenzyme M reductase I subunit gamma	1.8
Methyl-coenzyme M reductase I operon protein D	9.63
Methyl-coenzyme M reductase I operon protein C	unique to H_2_/CO_2_/CO
Tetrahydromethanopterin S-methyltransferase subunit H	1.83
**CODH/ACS related proteins**			
Acetyl-CoA synthase, subunit gamma	1.76
Acetyl-CoA synthase, subunit epsilon	4.62
Carbon monoxide dehydrogenase, iron sulfur subunit	3.48
CO dehydrogenase maturation factor	unique to H_2_/CO_2_/CO
**Acetate related genes**			
Predicted acetyl-coenzyme A synthetase	3.11


*A cutoff value of *p* < 0.05 was used to assess if proteins were more abundantly present. The complete proteomics dataset can be found in **Supplementary Table S1**.*

Polyferredoxin, belonging to the energy conserving hydrogenase (EcH), was more abundant in the presence of CO, but other subunits of this EcH were not clearly differentially present. Other hydrogenase related proteins, such as the HypE protein, involved in hydrogenase maturation, and subunits of the F420-reducing hydrogenase were also found to be more abundant in cultures grown with CO in the headspace. In presence of CO, several proteins related to redox stress were found in higher numbers: F420 oxidase, superoxide dismutase, superoxide reductase, and F390 synthetase (**Supplementary Table S1**). In addition to redox stress proteins, a predicted universal stress protein was found to be more abundant. Also a decrease in 30S and 50S ribosomal proteins was observed in presence of CO. Tuning down of ribosomal RNA and ribosomal proteins, is often observed as a response to different types of stress in *Escherichia coli* (Wagner, 1994), but also in other microorganisms this is observed (Abee and Wouters, 1999; van de Guchte et al., 2002). The decrease in ribosomal proteins in *M. marburgensis* in presence of CO could thus potentially be an additional indication of stress.

## Discussion

When growing on hydrogen, hydrogenotrophic methanogens generate methyl-H_4_MPT required in the final part of methanogenesis (Thauer et al., 2008). In addition, they can assimilate acetyl-CoA from methyl-H_4_MPT for biosynthetic purposes, by using the ACS. This requires CODH activity to reduce CO_2_ to CO, which is subsequently condensed with methyl-H_4_MPT and CoA-SH to form acetyl-CoA. For methanogens, the CODH complex is in almost all cases associated with an ACS complex (Techtmann et al., 2012), suggesting it mainly plays a role in the assimilatory metabolism.

### CODH/ACS Related Metabolism

The slight, but significant, increase in CO-oxidation activity observed in the CFE of H_2_/CO_2_/CO grown cultures compared to the CFE of H_2_/CO_2_ grown cells suggests that more CO-oxidizing enzymes are present. This is in accordance with the higher abundance of several CODH/ACS related proteins when *M. marburgensis* is grown in the presence of CO: the epsilon and gamma subunit of the main complex, the CODH maturation protein and a CODH related ferredoxin. Two CODH-related operons can be found in the genome of *M. marburgensis* (*cdh1* and *cdh2*; Liesegang et al., 2010). The *cdh2* operon codes for all the five subunits of a CODH/ACS, a CODH-like maturation factor and a CODH-related ferredoxin. Detection of the complete CODH/ACS complex under both of the tested conditions suggests that it is at least involved in assimilatory metabolism. Higher abundance of several CODH subunits, the CODH maturation factor and the CODH-related ferredoxin in the H_2_/CO_2_/CO condition could indicate that the complex is also involved in CO-oxidation, allowing transfer of electrons to other co-factors/proteins. Such an observation is also made in *M. acetivorans* of which the genome contains two CODH/ACS operons (*cdh1* and *cdh2*). Higher abundance of these CODH/ACS complexes was observed during proteomic analysis comparing CO-grown to acetate- or methanol-grown cultures. This suggests an important role for the CODH/ACS complex in CO-oxidation (Lessner et al., 2006; Rother et al., 2007). It has been shown later on that both Cdh isoforms of *M. acetivorans* are functional CODH/ACS complexes and that one isoform is sufficient for catabolic and anabolic functions (Matschiavelli et al., 2012). It is therefore likely that the CODH/ACS complex of *M. marburgensis* plays a main role in CO-oxidation.

The *cdh1* operon of *M. marburgensis* contains the sequence of one pseudo annotated CODH alpha subunit and a sequence coding for a HycB-like, ferredoxin-related, protein. The presence of the HycB gene next to the CODH sequence could indicate that this protein is involved in CO-oxidation. However, its function remains unclear as this protein is not detected in the H_2_/CO_2_ nor the H_2_/CO_2_/CO condition. The genome of *M. acetivorans* contains three other CODH sequences in addition to the two CODH/ACS operons: two monofunctional CODH related genes (*cooS1F* and *cooS2*) and one operon containing solely the alpha subunit of the CODH/ACS complex (*cdhA3*). Knockout studies showed that the two monofunctional CODHs are not essential for CO utilization, as CO was still utilized as a substrate in the knockout strains (Rother et al., 2007). These monofunctional CODHs were observed to have a function at elevated CO pressures as detoxification mechanism. The CdhA3 protein is suggested to play a role in CO sensing and is apparently not directly involved in CO oxidation (Matschiavelli et al., 2012). The Cdh1 of *M. marburgensis* was not detected in the conditions tested here, and thus no conclusions can be drawn on its function. However, as the CO concentrations used in this study are relatively low, it is possible this protein is involved in CO-detoxification at higher CO pressures, similar to the observation in *M. acetivorans*. However, a different role or non-functionality of this protein cannot be ruled out.

### Methyl-Branch Related Proteins

In addition to CODH/ACS related proteins, several enzymes involved in the methyl-branch of the reductive acetyl-CoA pathway were more abundant in cultures grown on H_2_/CO_2_/CO. In particular, formylmethanofuran dehydrogenase, acting in the formation of formylmethanofuran from CO_2_, and the F420-dependent methylene-H_4_MPT dehydrogenase, involved in generation of methyl-H_4_MPT from methylene-H_4_MPT, are significantly more abundant in incubations with CO (**Table 1**, **Figure 3**). Both proteins play a role in re-oxidation of co-factors, and their higher abundance might assist in countering the redox pressure of CO. Other proteins involved in the methyl-branch stay similar in abundance compared to incubations with H_2_/CO_2_ (**Figure 3**).

The higher abundance of several enzymes in the methyl-branch in H_2_/CO_2_/CO-grown *M. marburgensis* is expected to result in an increased flow through the pathway and thus requires up-regulation of subsequent pathways. This might explain the ‘switching-on’ of the acetate formation pathway and the increased production of enzymes involved in the final steps of methanogenesis (**Figure 3**). The observed acetate formation by *M. marburgensis* in presence of CO is supported by the finding of higher abundance of a predicted acetyl-coenzyme A synthetase (E.C. 6.2.1.1), theorized to be involved in acetate production and consumption. Proteomics data of *M. marburgensis* grown on H_2_/CO_2_/CO shows higher abundance of all subunits of methyl-coenzyme M reductase. Additionally, the H-subunit of tetrahydromethanopterin methyl-transferase is found to be more abundant. Studies on the H-subunit in *M. thermoautotrophicus* shows it catalyses the conversion of cob(I)alamin with CH_3_-H_4_MPT to methylcob(III)alamin, but lacks conversion activity of methylcob(III)alamin to methyl-CoM in absence of the rest of the complex (Hippler and Thauer, 1999). Other subunits of this complex are unchanged or even lowered in abundance in presence of CO (**Figure 3**). The membrane associated nature of this protein complex might have hindered accurate quantification, making it difficult to assess the relative abundance of the different subunits between the two conditions. This hinders the assessment of the role of the complex as a whole in the carboxydotrophic methanogenic metabolism of *M. marburgensis*.

Comparing the proteomics results obtained here with results reported for *M. acetivorans* is difficult as the native aceticlastic metabolism of *M. acetivorans* is different from the hydrogenotrophic metabolism of *M. marburgensis*. *M. acetivorans* cannot be grown on hydrogen and comparative proteomic studies with CO have been performed using acetate and methanol as alternative substrates (Lessner et al., 2006; Rother et al., 2007). A detailed comparison between the carboxydotrophic metabolism of *M. acetivorans* and *M. marburgensis* would therefore not be accurate.

### Limitations in Hydrogenotrophic Carboxydotrophic Methanogenic Metabolism

Carbon monoxide affects metalloproteins by interacting with their active-centers via back-bonding (Jeoung et al., 2014). Hydrogenases are considered CO-sensitive enzymes and H_2_ metabolism of several microbial strains is inhibited by CO (Daniels et al., 1977; Genthner and Bryant, 1982; Bertsch and Müller, 2015). However, [Ni–Fe]-hydrogenases are considered more robust and more resistant to CO-inhibition (Adams, 1990; De Lacey et al., 2007). Hydrogenases involved in methanogenesis are in general [Ni–Fe]-hydrogenases, except for the iron-only hydrogenase present in *M. thermoautotrophicus*, replacing the methylene-H_4_MPT reductase in nickel deprived conditions (Afting et al., 1998, 2000). Higher CO-pressures seem to inhibit the metabolism of both *M. thermoautotrophicus* and *M. marburgensis*, as can be seen from the slowing down in H_2_ consumption rate. However, the ability of both strains to co-utilize H_2_ and CO suggests hydrogenase inhibition is not the only mechanism of toxicity in these hydrogenotrophic methanogens.

Carbon monoxide has a low redox-potential (*E*^0′^ = -520 mV), which can be seen as a major difficulty for the methanogens. Generation of ferredoxin by hydrogenotrophic methanogens is in general done via reverse proton transport via the EcH or a bifurcation reaction performed by a F420-non-reducing hydrogenase (Buckel and Thauer, 2013). The low redox potential of CO does, however, allow for direct reduction of ferredoxin via CODH, making reduced ferredoxin more accessible in the cell. This lowers the *Fd*_ox_/*Fd*_red_ ratio in the cell, lowering the electron potential of the ferredoxin couple. Judging from proteomics data, several redox response systems are activated. However, many of these, such as the superoxide dismutase and F420-oxidase, are normally a response to oxidized compounds (e.g., oxygen), which is the opposite of the more reduced environment created by CO. It might be that these genes respond to redox stress in general or are regulated by universal stress proteins. Additionally, the F390-synthetase, involved in redox sensing was found to be more abundant. In *M. thermoautotrophicus*, this system was found to react on changes in redox potential, regulating the expression of MCR-I, F420-dependant MDH, and the F420-reducing hydrogenase (Vermeij et al., 1997). The CFE of H_2_/CO_2_/CO grown cells showed more endogenous activity when compared to H_2_/CO_2_ grown cells. This could indicate more reducing equivalents are present in the cells grown in presence of CO. The overall increase in abundance of proteins involved in redox stress and co-factor regeneration, and the higher endogenous reducing activity suggest that *M. marburgensis* cells exposed to CO are subjected to changes in redox balance.

**FIGURE 4:**
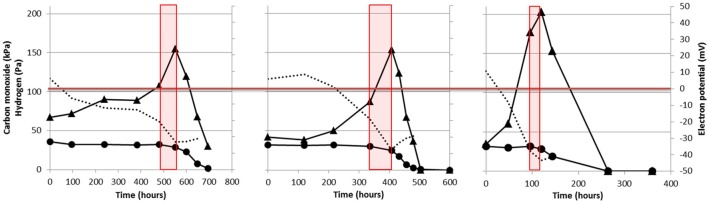
**Thermodynamic analysis of the reaction catalyzed by the F420-non-reducing hydrogenase in *M. marburgensis*.** The used dataset is the same as the one displayed in **Figure 2**. H_2_ pressure in Pa is given by the black triangles and the carbon monoxide pressure in kPa is given by the black circles. The estimated difference between the two reactions catalyzed by the bifurcating F420-non-reducing hydrogenase is indicated by the dotted black line. Red boxes indicate the phase where methanogenesis is initiated. The last time point is not assessed as the gasses had reached a pressure which could not be determined accurately.

When grown solely on CO, *M. marburgensis* shows production of H_2_ from the moment incubation is started (**Figure 2**). Methanogenesis is not started instantly, and CO appears to be solely used for H_2_ production. Methanogenesis initiates only after H_2_ has accumulated in the headspace (**Figures 2** and **4**) and, from that point on, quickly co-utilizing H_2_ and CO. These data suggest that a minimal amount of H_2_ is required to operate the carboxydotrophic methanogenic metabolism. We theorize this is related to the properties of the bifurcating F420-non-reducing hydrogenase required to regenerate CoM-SH and CoB-SH. Feasibility of the overall bifurcating reaction was estimated by calculating the potential difference between the two separate reactions catalyzed (Eqs 1 and 2). The following assumptions were made during calculation: (I) pH is assumed to be 7 and temperature 338 K, (II) the ferredoxin couple is assumed to have a standard electron potential of -500 mV under physiological conditions (Thauer et al., 2008), but is assumed to approach the electron potential of the CO/CO_2_ couple at the respective partial pressures (assessed via Nernst equation). Similarly the electron potential of H_2_ is assumed to be similar to its partial pressure, (III) the CoM–CoB couple is assumed to have an electron potential of -140 mV, and (IV) the bifurcation reaction is considered feasible if the sum in potential of reaction 1 and 2 has a negative value.

$$H_{2}+Fd\to 2H^{+}Fd^{2-}\,E^{0^{\prime }}=+86\,m\,V$$

$$CoM-CoB+H_{2}\to CoM-SH+CoB-SH\,\;E^{0^{\prime }}=-274\,\;m\,\;V$$

H_2_ has to drive the reduction of both CoM–CoB and ferredoxin in the reaction catalyzed by the F420-non-reducing hydrogenase. However, as the electron potential of ferredoxin is expected to be lowered in presence of CO, and H_2_ is almost absent in the cultures, the overall reaction becomes less exergonic. When assessing the difference between the two separate reactions, it is observed that the overall reaction is not favored under the starting conditions of incubation (**Figure 4**, dotted lines). Under these conditions the CoM–SH and CoB–SH cannot be replenished, potentially blocking methanogenesis. As more H_2_ is generated, via the EcH complex, the reaction becomes more favorable, eventually allowing to start methanogenesis (**Figure 4**). Inhibition of hydrogenases or an irreversible reduced state of the cell at higher CO pressures could prevent H_2_ production and consumption, preventing the methanogenic metabolism from starting. The proposed limitation of the F420-non-reducing hydrogenase might explain why hydrogenotrophic methanogens as *M. marburgensis* and *M. thermoautotrophicus* can only utilize CO as a sole substrate up to 50 kPa, whereas *M barkeri* and *M. acetivorans*, which do not employ a bifurcating F420-non-reducing hydrogenase (Thauer et al., 2008), can perform carboxydotrophic growth using >100 kPa and >150 kPa CO, respectively. The CO metabolism of hydrogenotrophic methanogens is thus potentially limited by their F420-non-reducing hydrogenase, and this would explain the observation that their efficiency of carboxydotrophic growth is strongly connected to the availability of hydrogen.

## Conclusion

The hydrogenotrophic methanogen *M. marburgensis*, was found to grow methanogenically on H_2_/CO_2_/CO or CO alone. CO could be used as a sole substrate up to 50 kPa and its consumption was stimulated in the presence of hydrogen. Proteomic analysis indicates higher abundance of CODH/ACS related proteins and enzymes of the methyl-branch in presence of CO. Most of the abundant proteins in cultures grown in the presence of CO are involved in redox reactions, potentially required to counter the strong reducing capacity of CO. Additionally, the pathway toward acetate production was found up-regulated, which explains formation of small amounts of acetate as an end-product in presence of CO. The ability to utilize H_2_ in the presence of CO suggests that hydrogenase inhibition is not the main mechanism of toxicity in hydrogenotrophic methanogens. The requirement of small amounts of hydrogen, before methanogenesis with CO could start, suggests that this is an essential intermediate in the methanogenic metabolism. The F420-non-reducing bifurcating hydrogenase is a likely candidate for CO inhibition as low H_2_ pressures potentially can cause this reaction to become unfavorable, blocking the methanogenic metabolism.

## Author Contributions

MD: Experimental work, data analysis, drafting and writing manuscript. RP: Experimental work and data analysis. HW:Proteomics and data analysis. AS: Experimental design and critical revision of the manuscript. DS: Experimental design, data analysis, and writing the manuscript.

## Conflict of Interest Statement

The authors declare that the research was conducted in the absence of any commercial or financial relationships that could be construed as a potential conflict of interest.

## Supplementary Material

**TABLE S1 | Comparative proteomic analysis of H_2_/CO_2_ vs. H_2_/CO_2_/CO grown cells of *Methanothermobacter marburgensis*.**
